# Grease in the Nucleus: Insights into the Dynamic Life of Nuclear Membranes

**DOI:** 10.1007/s00232-022-00272-8

**Published:** 2022-11-04

**Authors:** Deepak Anand, Arunima Chaudhuri

**Affiliations:** 1 The Microbiology Group, Department of Biology, Biology Building, Lund University, Sölvegatan 35, 223 62 Lund, Sweden; 2 Department of Microbiology, Immunology and Glycobiology, Institute of Laboratory Medicine, Lund University, Sölvegatan 19, 223 62 Lund, Sweden

**Keywords:** Nuclear membrane, Lipids, Proteins, Nuclear blebs, Nucleoplasmic reticulum

## Abstract

Nucleus is at the center stage of cellular drama orchestrated in the life of a cell and the nucleoplasm is surrounded by a double membranous compartment constituting the Nuclear membrane/envelope (NE) that separates it from the cytoplasm in nucleated cells. The initial understanding of the NE was that of a border security entity between the nucleus and the cytoplasm, separating gene regulation and transcription in the nucleus from translation in the cytoplasm. However, the discovery of a wide array of inherited diseases caused by mutations in genes encoding proteins that reside or interact with NE diverted the interest into deciphering the lipid-protein-rich environment of the NE. Today, the NE is considered a dynamic organelle which forms a functional linkage between the nucleus and the rest of the cell. The exposure of NE to constant mechanical constraints by its connectivity to the large polymer network of the lamina and chromatin on one side, and to the cytoskeleton on the other side results, in a variety of shape changes. We discuss two such deformation, the formation of nuclear blebs and nucleoplasmic reticulum (NER). Although the protein and the lipid composition of NE comprises a small fraction of the total lipid-protein load of the cell, the ability to define the lipid-protein composition of Inner nuclear membrane (INM) and Outer nuclear membrane (ONM) with precision is crucial for obtaining a deeper mechanistic understanding of their lipid-protein interaction and the various signaling pathways that are triggered by them. In addition, this allows us to further understand the direct and indirect roles of NE machinery in the chromosomal organization and gene regulation.

**Graphical Abstract:**

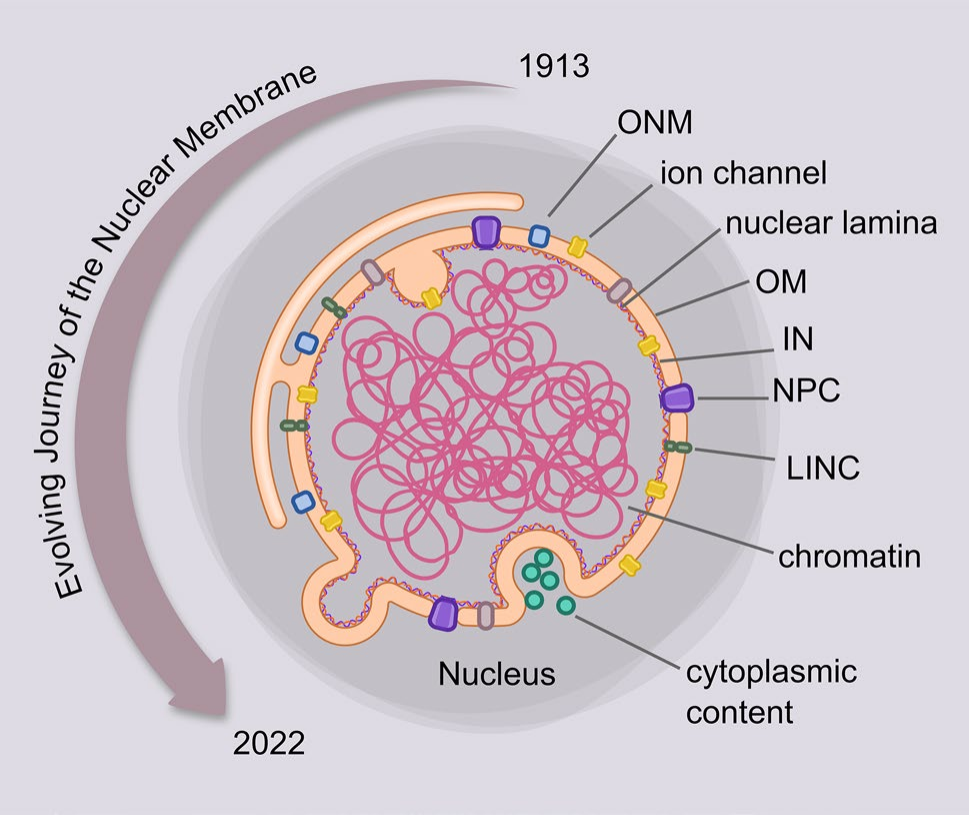

## Introduction

**Fig. 1:**
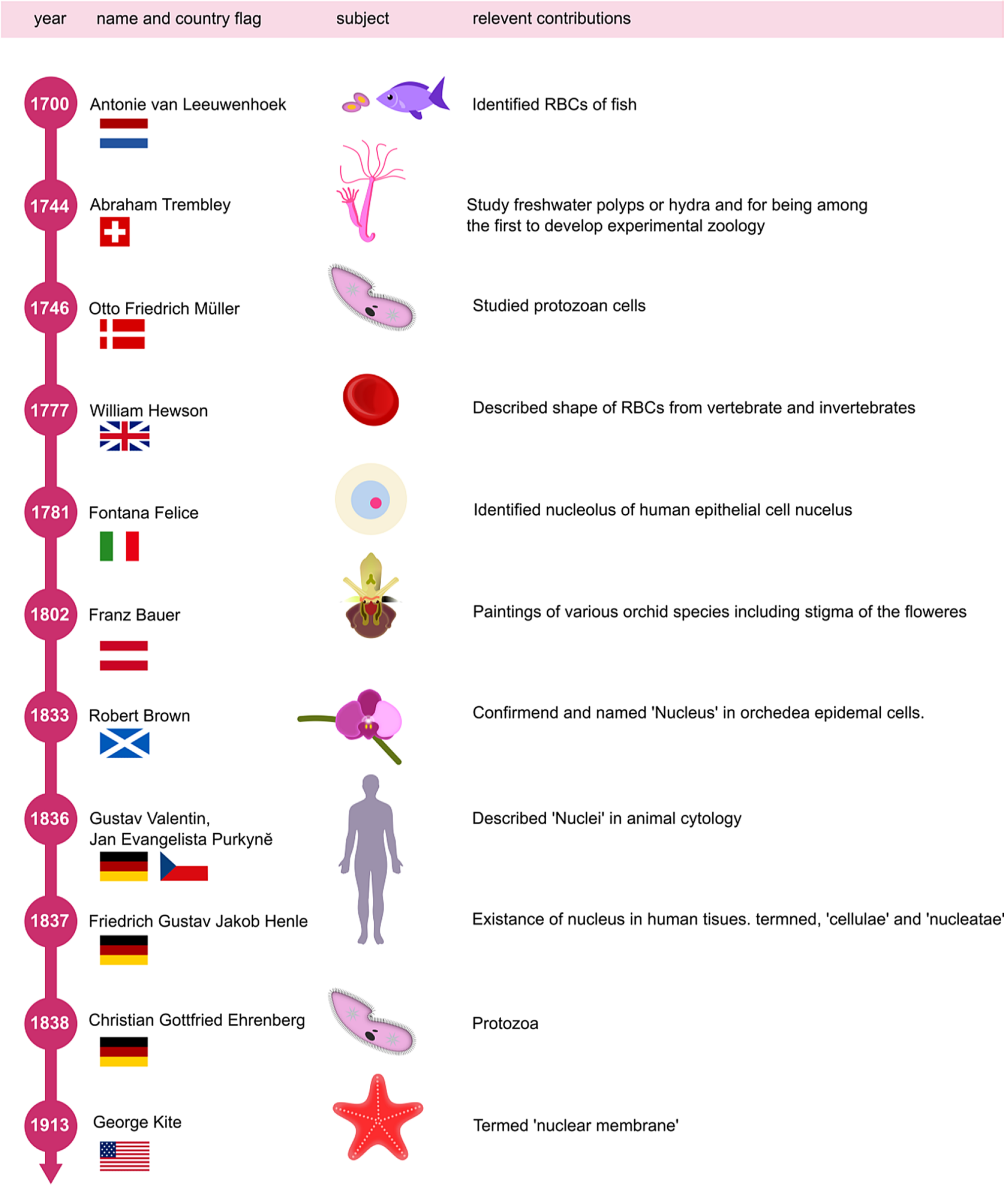
Timeline of the early observations marking the discovery of the nucleus and the nuclear membrane. First observation of the nucleus was reported by Leeuwenhoek in fish erythrocytes, followed by the observations of Trembley, Müller, Ehrenberg, Hewson, Fontana and Bauer. Bown, Valentin and Henle recognized the quasi universality of nucleus in plants and animals. Later in twentieth century, Kite used the term nuclear membrane for the first time to describe a membranous structure around the nucleus. Information obtained from Osorio and Gomes, (2013) and Kite (1913)

Nucleus is at the center stage of cellular drama orchestrated in the life of a cell and the universality of its presence in plants and animals was first proposed in the 1830s by Brown in plants; and Valentin and Henle in animals (Osorio and Gomes, 2013). The history of the initial observations of the nucleus is extremely fascinating and has been covered in detail elsewhere (Osorio and Gomes, 2013). We have provided a schematic of the timeline of the scientists who contributed to this initial journey of observing the nucleus (Fig. 1). The nucleoplasm is surrounded by a double membranous compartment constituting the Nuclear membrane/envelope (NE) that separates it from the cytoplasm in nucleated cells. The initial understanding of the NE since its discovery in 1913 (Kite 1913) has been that of a border security entity between the nucleus and the cytoplasm, separating gene regulation and transcription in the nucleus from translation in the cytoplasm. The switch from the chromatin-centric understanding of the nucleus to unravelling the lipid-protein-rich environment of the NE was spearheaded by the discovery that mutations in genes encoding its protein components cause a wide array of inherited diseases often referred to as laminopathies or nuclear envelopathies. These pathologies include movement disorders and myopathies (Dauer and Worman 2009; Meinke and Schirmer 2016), aging-related diseases causing reduced life span and progeria (Kubben and Misteli 2017; Fichtman et al 2019), lethal defects in the embryo (Turner and Schlieker, 2016), and lipodystrophies (Shackleton et al. 2000). Disruption of NE stability is also common in cancer cells causing DNA damage, cancer-relevant chromosomal rearrangements, and the initiation of pro-inflammatory pathways (Lim et al. 2016; Umbreit and Pellman 2017; Hatch 2018; Selezneva et al. 2022), underscoring the need to unravel its organization and dynamics.

**Fig. 2:**
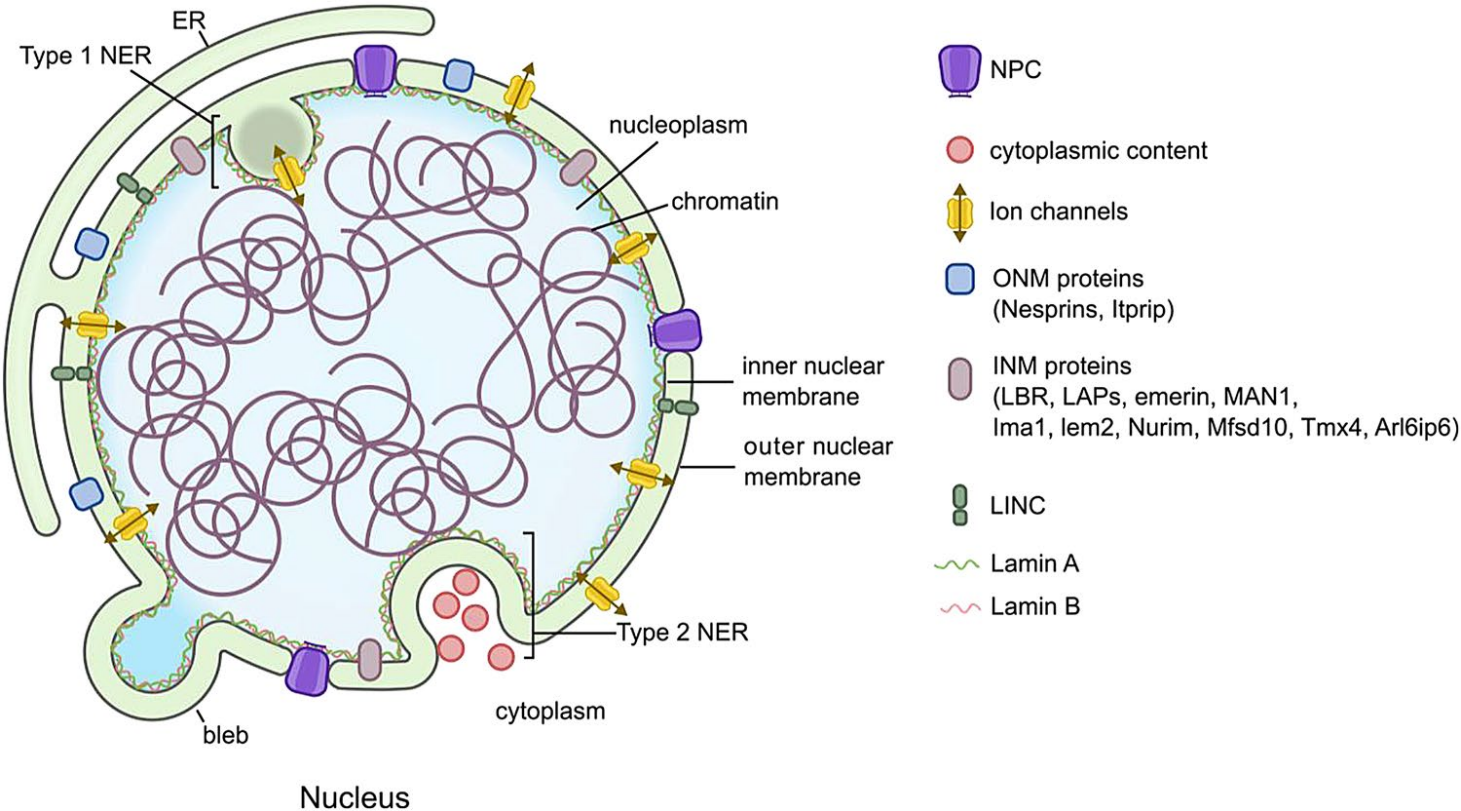
A schematic representation of NE showing the structure and dynamic deformations. NE is a dynamic double membrane organelle which forms a functional linkage between the nucleus and the rest of the cell. The two membranes are bridged by NPCs and the LINC complexes. Two types of deformations observed in the NE are the outward projecting blebs and inward projecting invaginations called NER

Today, the NE is considered a dynamic organelle which forms a functional linkage between the nucleus and the rest of the cell (Fig. 2). Biological mass-spectrometry techniques and analysis algorithms have expanded our knowledge of the nuclear protein repertoire. However, understanding its lipid-protein composition and their interaction remains challenging owing to (i) purifying nuclear membrane without Endoplasmic reticulum (ER) contamination is very difficult and (ii) due to the proximity of the two membranes of NE. Immunogold-label Electron microscopy (EM) remains the method of choice for determining the location of NE residing proteins with precision. In recent years, techniques such as rapamycin trapping, using spit-GFP constructs, metal-induced energy transfer, ensemble Fluorescence recovery after photobleaching (FRAP) and super-resolution microscopy have been adapted to address the differential understanding of Inner nuclear membrane (INM) *vs* outer nuclear membrane (ONM) (Tingey et al. 2019). In this mini-review, we summarize the current understanding of the lipid-protein machinery of the NE and the dynamic deformations that occur in the NE in physiological and pathological states.

## Protein Machinery of NE

Protein machinery of NE contributes to 1% (278 proteins) of all human proteins experimentally detected by the Human Protein Atlas with 238 of them having multiple locations outside of NE. Interestingly, 10% of eukaryotic transmembrane proteins are found to be residing in NE (Mudumbi et al. 2020). The ONM is continuous with the rough ER membrane, and they are generally similar in protein content with only a few proteins, namely nesprins and Itprip, preferentially concentrated in the ONM (Méndez-López et al. 2012; Cheng et al. 2019). In contrast, the protein machinery of the INM is still poorly understood. A comprehensive proteomic study identified 13 knowns and 67 putative proteins concentrated in the INM (Schirmer et al. 2003; Méndez-López et al. 2012; Cheng et al. 2019). The few signature proteins of INM include the integral proteins Lamin B receptor (LBR), Lamina-associated polypeptides (LAPs), emerin and MAN1 (also known as LEMD3), Ima1 (Ima1 present in fission yeast is a homologue of the human Samp1 and rat NET5 proteins) and Lem2 (Collas et al. 2000; Tange et al. 2016). Newly identified members of INM include Nurim, Mfsd10, Tmx4, and Arl6ip6 (Chen et al. 2012; Cheng et al. 2019). A recent review covered the current understanding of the diverse cellular functions of INM proteins in detail (Pawar and Kutay 2021).

The two membranes of the NE are separated by 30–50 nm, and NE bridges composed of Sad1 and UNC-84 (SUN) proteins in INM and Klarsicht, Anc-1, and Syne homology (KASH) proteins in ONM have been proposed to set and regulate nuclear envelope spacing (Sosa et al. 2012; Cain et al. 2014). These proteins also constitute the Linker of the nucleoskeleton and cytoskeleton (LINC) complex, which physically connects the nucleus and plasma membrane via the actin cytoskeleton to perform diverse functions including mechano-transduction from the extracellular environment to the nucleus (Ueda et al. 2022).

In addition to the LINC complex, large macromolecular assemblies (~ 100 mDa) constituting NPCs bridge the two membranes of NE. Nuclear pore complexes (NPCs) mediate molecular flux between the nucleus and the cytoplasm and are built by ~ 1000 protein subunits called Nucleoporins (NUP). The biogenesis of NPCs during the interphase of the cell cycle and their insertion in NE by fusion between the INM and ONM has been covered in detail elsewhere (Rothballer and Kutay 2013). NPCs have been traditionally studied as a selective pore allowing the trafficking of molecules through the double lipid bilayers, recent studies highlight its role in chromosomal organization and gene regulation, as it can interact with the genomic region enhancers and super-enhancers (Lin et al. 2019; Pascual-Garcia et al. 2019). Polar molecules, ions and macromolecules are allowed to pass between nucleoplasm and cytoplasm through the NPCs (Hampoelz et al. 2019).

Another important protein constituent of NE are the ion channels which have been discovered using genetic, immunological, pharmacological, and electrophysiological approaches (Matzke et al. 2010). The most well understood ion channels in NE are the Ca^2+^ channels in animals and plants (Bootman et al., 2009; Oliveira et al. 2014; Secondo et al. 2020; Pirayesh et al. 2021). The Ca^2+^ channels found in the INM include Inositol (1,4,5)-trisphosphate receptor (IP_3_R), Ryanodine receptor (RyR), and Nicotinic acid-adenine dinucleotide (NAADP) receptors. The ONM resident Ca^2+^ channels are IP_3_R, Ca^2+^-ATPases and inositol 1,3,4,5-tetrakisphosphate-operated Ca^2+^ channels (Becchetti 2011). In addition, chloride channels (Gururaja et al. 2020), potassium channels (Jang et al. 2015) Ca^2+^ -ATPase (Gerasimenko et al. 1995) and Na^+^/Ca^2+^ exchangers (Secondo et al. 2020) are also present in the NE.

The INM is lined by the Nuclear lamin (NL), belongs to type V intermediate filament proteins, and is divided into two subtypes, the A and B. The gene LMNA is spliced in two isoforms, the longer version encoding the protein Lamin A and the shorter isoform generating the Lamin C protein. Two different genes (LMNB1 and LMNB2) are responsible for encoding Lamin B1 and B2 proteins. Details of NL and their post-translational modifications and their active role in signaling have been covered elsewhere (Gauthier et al. 2021).

## Grease of the NE: Lipids in Action

NE constitute a very small fraction (< 1%) of the total membrane content of the cell (Milo and Phillips 2015) and its composition has been analyzed by mass-spectrometry and multidimensional NMR (^31^P and ^1^H) (van Meer et al. 2008; Dazzoni et al. 2020a). NE lipid extract demonstrated a complex mixture of phospholipids with different fatty acyl chain lengths varying between 30 and 38 carbon atoms (two chains summed up) associated predominantly with Phosphatidylcholine (PC) head group (van Meer et al. 2008; Dazzoni et al. 2020a). Negatively charged lipids were also observed with an abundance of Phosphatidylinositol (PI), associated with chain length varying between 36 and 38 carbon (Dazzoni et al. 2020a). NE lipid extracts analyzed by ^1^H–,^13^C– and ^31^P-NMR showed the presence of cholesterol in addition to PE and PI (Dazzoni et al. 2020b). The presence of elevated levels of unsaturated fatty acid chains (with one to two double bonds per lipid species) accentuated the fluidity and elasticity of NE relative to plasma membrane (Dazzoni et al. 2020a, b). Despite the enhanced membrane fluidity, the INM and the ONM are only permeable to small non-polar molecules and sustain nucleoplasm membrane potential approximately − 15 mV with respect to the cytoplasm (Loewenstein and Kanno 1963; Mazzanti et al. 2001).

Recent studies have highlighted that although NE is not a major site of lipid synthesis compared to the ER, changes in NE lipid composition occur as an adaptive stress management response to the maintain the integrity of NE. Studies in yeast, fly and mammalian cells have shown that de novo PC synthesis can take place to relieve the curvature elastic stress and NE breakdown (Haider et al. 2018). Accumulation of very-long-chain fatty acids or phytoceramides by the action of NE resident very-long-chain fatty acid elongase Elo2 has been observed in yeast to prevent lethal defects associated with Lem2 and Bqt4 knockouts, which are conserved nuclear membrane proteins (Kinugasa et al. 2019). Interestingly, targeting ceramide synthesis suppresses nuclear abnormalities and improves the proliferation of aneuploid cells in yeasts and patients associated with Down syndrome (Hwang et al. 2019). In addition, a sphingolipid hydrolase (Smpd4) that releases ceramide spatially localizes to NPCs, suggesting a potential local role for sphingolipids and their precursors at the NE (Cheng et al. 2019).

## How is Identity of the NE Maintained?

How is identity of the NE maintained despite the ONM being continuous with the ER? The code to understand the sorting of lipids and proteins within the ER/NE membranes and how NE maintains its identity is still not cracked. The ability to retain proteins in the INM has been linked to their affinity for nuclear components *e.g.,* for LBR, SUN2, LAP2β and the phenomenon termed as “diffusion and retention” (Ungricht et al. 2017). Interestingly, many INM proteins contain NLS-like sequences (Lusk et al. 2007) and Kutay and group proposed that these could function as nuclear retention motifs, *e.g.*, as part of DNA-binding domains (LaCasse and Lefebvre 1995; Cokol et al. 2000).

Recent evidence suggests that lipids produced in the ER are harnessed to remodel nuclear membranes (Barger et al. 2022).

Is there an asymmetry in the lipid composition of INM *vs* ONM? Specific lipids residing in INM have been shown to support viral proliferation (Marschall et al. 2011), NE dynamics (Hatch and Hetzer 2014), de novo lipid synthesis (Haider et al. 2018; Romanauska and Kohler 2018) and NPC biogenesis (Drin et al. 2007) underscoring the existence of mechanisms that differentially enrich and regulate specific lipid species at the INM. Currently limited knowledge exists to understand these processes and potential mechanisms that drive lipid asymmetry at the NE and lead to NE remodeling, despite the direct continuity of the lipid bilayers of the NE and ER. Two pathways have been proposed to attain this asymmetry: (1) the presence of a physical barrier that reduces the timescale of lateral diffusion for specific lipids from one area (peripheral ER/ONM) to the other (INM) and (2) differential spatial restriction of synthetic enzymes to generate a continuum of concentrations high in one area (*e.g.*, peripheral ER) relative to the other (*e.g*., INM). These pathways have been discussed in detail in other recent reviews (Bahmanyar and Schlieker 2020; Barger et al. 2022).

## NE Membrane Deformations

The exposure of NE to constant mechanical constraints by virtue of its connectivity to the large polymer network of the laimina and chromatin on one side, and to the cytoskeleton on the other side results, in a variety of shape changes. A recent review covered the mechanisms and functions of NE remodeling in exquisite detail (Ungricht and Kutay 2017). Here we discuss, two main types of NE deformations that have been observed: the outward projecting nuclear blebs and the inward projecting nuclear invaginations constituting the nucleoplasmic reticulum (NER).

Nuclear blebs are formed when the double NE separates from the lamina and chromatin, inflates and forms a round protrusion containing nucleoplasm which is not retracted like the plasma membrane blebs and possesses a high likelihood of bursting (Srivastava et al 2021). Nuclear blebs form as spherical protrusions filled with nucleoplasm and devoid of chromatin and are commonly associated with sites of local lamina weakness (Charras et al. 2008; Wiggan et al. 2017; Shah et al 2017). The membrane rupture occurs systematically in these swollen blebs, measuring ~ microns in diameter, as a consequence of internal pressure mounted by translocation of nucleoplasm inside the blebs (Srivastava et al 2021). However, these membrane rupture events are followed by rapid repair (Raab et al. 2016; Earle et al. 2020). Some of these blebs are associated with chromatin herniation, which results in protrusion of the chromatin through the local rupture of the lamina at the base of the bleb. A new lamina eventually reforms on the surface of the herniated chromatin as the herniation is not retracted, leading to a long-term nuclear shape alteration after the resealing of the envelope (Charras et al. 2008). The uncontrolled exchange between the nuclear interior and cytoplasm occurs in the nuclear blebs and these sites prime DNA damage (Shah et al. 2017). Nuclear blebs have been observed in several cancerous cells such as in monoblasts of acute monocytic leukaemia (McDuffie 1967), Burkitt lymphoma cells (Epsteln et al. 1965; Achong and Epstein 1966), and anaplastic giant cell carcinoma of the thyroid (Caryso et al 2011). Accumulation of these blebs has been observed in laminopathies such as premature Hutchinson–Gilford progeria syndrome and Emery–Dreifuss muscular dystrophy (Lattanzi et al. 2016). Interestingly, nuclear blebbing has also been observed in developing human and guineapig thymocytes (Törö and Oláh 1966; Sebuwufu 1966) suggesting NE plasticity during functional development and differentiation of the cells.

The continuity of NE is interrupted by invaginations that reach deep within the nucleoplasm and such a complex branched network of invaginations has been defined as NER (Malhas et al. 2011). Lipids extracts from NER were at least two orders of magnitude more elastic than the classical plasma membrane suggesting a physical explanation for the formation of NER (Dazzoni et al. 2020b). Morphological comparisons with the ER paved the nomenclature of these widespread intra-nuclear invaginations as NER (Echevarría et al. 2003; Fischer et al. 2003). NER structures are classified into 2 main classes: Type I invaginations where the INM alone invaginates into the nucleoplasm, whereas type II where both the INM and ONM enter the nucleoplasm allowing the presence of a cytoplasmic core (Drozdz, and Vaux 2017). The function of NER in physiology and pathology has been covered in detail in recent reviews (Drozdz, and Vaux 2017; Stiekema et al. 2022).

## Dynamic Nature of NE

NE is a highly dynamic (temporally) organelle based on its ability and need to deform at small and large length scales. However, sparse studies exist which have looked at the mobility of lipids and protein in NE. Spectrometry analysis of NE of HEK 293 T cells showed that these membrane consist of PC (63%), PE (9%), SM (4%) and PI (12%) (Dazzoni et al. 2020a). Interestingly, cholesterol is also thought to be an important lipid in these membrane although its exact amount is still debatable. Measurements using solid-state NMR showed that the NE lipids derived from these cells are 100 times more elastic than plasma membranes (Dazzoni et al. 2020b). The abundance of unsaturated in the fatty acyl chains of PI coupled with its negative charge is thought to be balancing factor counteracting the rigidifying effect of cholesterol in NE (Dazzoni et al. 2020a).

FRAP based studies have shown that GFP-tagged emerin, MAN1 and LBR-are less mobile in the nuclear envelope than in the ER (Ellenberg et al. 1997; Östlund et al., 1999; Wu et al., 2002). Diffusional mobility (D) of emerin was decreased in INM (D = 0.10 ± 0.01 µm^2^/second) compared to the ER membrane (D = 0.32 ± 0.01 µm^2^/second). MAN1 also demonstrated a lower mobility in the INM (0.12 ± 0.02 µm^2^/second) relative to the ER pool (D ~ 0.28 ± 0.04 µm^2^/second). Early studies addressing the mobility of proteins in NE membranes using isolated nuclei chemically modified with citraconic acid showed that D for proteins in the INM bound by the fluorescently labeled lectin wheat germ agglutinin was 0.039 mm^2^/s (Schindler et al., 1984). Enhanced mobility of GFP-tagged emerin and MAN1, but not LBR, was observed in embryonic fibroblasts from lamin A knockout (Lmna^−/−^) mice implying that emerin and MAN1 are partly retained in the INM by binding to A-type lamins, while LBR depends on other binding partners for its retention.

## Conclusion

This is a very exciting time in membrane biology, as the landscape of our understanding of membranes is beginning to expand from the classical plasma membrane to the intracellular organelles. NE is at the center stage of this revolution as we are understanding its many facets with the improvement of technology available to tease out its organization and dynamics. The challenges to overcome include the ability to define the lipid-protein composition of INM and ONM with precision to address the dynamic lipid-protein interaction in these complex membranes. Purifying and isolating nuclear membrane without ER contamination is still considered as the holy grail of the field. The traditional method for nuclei isolation involves the use of non-ionic detergent (Lee et al., 2010), which suffer from the tendency to cause unwanted nuclear aggregation and disrupt the NE resulting in stripping/loss of of some NE proteins in addition to leakage of nuclear matrix materials. Detergent free methods have been described that purify the nucleus without these side effects (Blobel and Potter 1966; Eski et al., 2020).

Solving the challenges mentioned above is quintessential for obtaining a deeper mechanistic understanding of the various signaling pathways that are triggered by NE residents or interacting protein-lipid machinery. In addition, this allows us to further understand its direct and indirect roles in the chromosomal organization and gene regulation.

## Data Availability

All data generated or analyzed during this study are included in this published article which are listed in the references.

## Abbreviations

NE: Nuclear membrane/envelope
ER: Endoplasmic reticulum
EM: Electron microscopy
INM: Inner nuclear membrane
ONM: Outer nuclear membrane
LBR: Lamin B receptor
LAPs: Lamina-associated polypeptides
SUN: Sad1 and UNC-84
KASH: Klarsicht, Anc-1, and Syne homology
LINC: Linker of the nucleoskeleton and cytoskeleton
NPC: Nuclear pore complexes
NUP: Nucleoporins
NL: Nuclear lamin
PC: Phosphatidylcholine
PI: Phosphatidylinositol
NER: Nucleoplasmic reticulum
FRAP: Fluorescence recovery after photobleaching
D: Diffusional mobility
GFP: Green fluorescent protein
IP_3_R: Inositol (1,4,5)-trisphosphate receptor
RyR: Ryanodine receptor
NAADP: Nicotinic acid-adenine dinucleotide
RBC: Red blood cells
